# The power of FDG-PET to detect treatment effects is increased by glucose correction using a Michaelis constant

**DOI:** 10.1186/2191-219X-2-35

**Published:** 2012-06-27

**Authors:** Simon-Peter Williams, Judith E Flores-Mercado, Andreas R Baudy, Ruediger E Port, Thomas Bengtsson

**Affiliations:** 1 Department of Biomedical Imaging, Genentech, Inc., 1 DNA Way, South San Francisco, CA, 94080, USA; 2 Department of Pharmacokinetics and Pharmacodynamics, Genentech, Inc., South San Francisco, CA, 94080, USA; 3 Department of Biostatistics, Genentech, Inc., South San Francisco, CA, 94080, USA

**Keywords:** FDG-PET, Glucose correction, Michaelis-Menten, Response to treatment, Glucose bias

## Abstract

**Background:**

We recently showed improved between-subject variability in our [^18^F]fluorodeoxyglucose positron emission tomography (FDG-PET) experiments using a Michaelis-Menten transport model to calculate the metabolic tumor glucose uptake rate extrapolated to the hypothetical condition of glucose saturation: $MR_{\mathrm{gluc}}^{\max}=K_{i}*(K_{M}+[glc])$, where *K*_i_ is the image-derived FDG uptake rate constant, *K*_M_ is the half-saturation Michaelis constant, and [glc] is the blood glucose concentration. Compared to measurements of *K*_i_ alone, or calculations of the scan-time metabolic glucose uptake rate (MR_gluc_ = *K*_i_ * [glc]) or the glucose-normalized uptake rate (MR_gluc_ = *K*_i_*[glc]/(100 mg/dL), we suggested that $MR_{\mathrm{gluc}}^{\max}$ could offer increased statistical power in treatment studies; here, we confirm this in theory and practice.

**Methods:**

We compared *K*_i_, MR_gluc_ (both with and without glucose normalization), and $MR_{\mathrm{gluc}}^{\max}$ as FDG-PET measures of treatment-induced changes in tumor glucose uptake independent of any systemic changes in blood glucose caused either by natural variation or by side effects of drug action. Data from three xenograft models with independent evidence of altered tumor cell glucose uptake were studied and generalized with statistical simulations and mathematical derivations. To obtain representative simulation parameters, we studied the distributions of *K*_i_ from FDG-PET scans and blood [glucose] values in 66 cohorts of mice (665 individual mice). Treatment effects were simulated by varying $MR_{\mathrm{gluc}}^{\max}$ and back-calculating the mean *K*_i_ under the Michaelis-Menten model with *K*_M_ = 130 mg/dL. This was repeated to represent cases of low, average, and high variability in *K*_i_ (at a given glucose level) observed among the 66 PET cohorts.

**Results:**

There was excellent agreement between derivations, simulations, and experiments. Even modestly different (20%) blood glucose levels caused *K*_i_ and especially MR_gluc_ to become unreliable through false positive results while $MR_{\mathrm{gluc}}^{\max}$ remained unbiased. The greatest benefit occurred when *K*_i_ measurements (at a given glucose level) had low variability. Even when the power benefit was negligible, the use of $MR_{\mathrm{gluc}}^{\max}$ carried no statistical penalty. Congruent with theory and simulations, $MR_{\mathrm{gluc}}^{\max}$ showed in our experiments an average 21% statistical power improvement with respect to MR_gluc_ and 10% with respect to *K*_i_ (approximately 20% savings in sample size). The results were robust in the face of imprecise blood glucose measurements and *K*_M_ values.

**Conclusions:**

When evaluating the direct effects of treatment on tumor tissue with FDG-PET, employing a Michaelis-Menten glucose correction factor gives the most statistically powerful results. The well-known alternative ‘correction’, multiplying *K*_i_ by blood glucose (or normalized blood glucose), appears to be counter-productive in this setting and should be avoided.

## Background

Quantitative ^18^F]fluorodeoxyglucose positron emission tomography (FDG-PET) is increasingly relied upon to measure pharmacodynamic responses in controlled trials, bringing a greater need for accurate and reproducible scans to minimize the number of subjects needed for a successful trial. Glucose levels have long been recognized as a factor modulating FDG uptake [1-8]; but even so, there has been some debate regarding how best to compensate for changing glucose levels when comparing scans. Some investigators have eschewed glucose corrections altogether after observing increased rather than decreased statistical noise in ‘corrected’ PET measurements, attributing this, perhaps, to error in the glucose measurement itself [9,10]. However, avoiding glucose correction poses a conundrum of interpretation when a treatment may induce a systematic change in blood glucose levels. Such treatments are known, and FDG-PET may be used to assess their impact; they include some potentially important new drugs still under clinical investigation, such as certain Akt and PI3K inhibitors [11,12].

The seminal work of Sokoloff et al. [13] described the Michaelis-Menten kinetics of glucose and tracer transport and showed how the radioactive tracer uptake rate constant (*K*_i_) could be used to estimate the tissue glucose uptake in physiological units, i.e., the metabolic rate of glucose (MR_gluc_ = *K*_i_*[glc]/LC μmol glucose per 100 g tissue per min). Under steady-state conditions, the half-saturation Michaelis constants (*K*_M_) and the maximal velocities (*V*_max_) for tracer and glucose are factored into the lumped constant (LC) which summarizes the differential properties of tracer and glucose. Scans obtained under different blood glucose levels will almost inevitably indicate different metabolic rates of glucose, and one must decide how to detect changes in tumor glucose metabolism that are not merely due to changes in blood glucose.

We recently demonstrated [14] that in untreated animals, both tumor *K*_i_ values and MR_gluc_ values were, on the average, strongly correlated with blood glucose, showing that an appropriate form of blood glucose correction might facilitate the identification of treatment effects under changing glucose conditions. We sought to understand this glucose effect so that an appropriate compensating correction could be made, expecting that this would improve the power to detect treatment effects.

The Michaelis-Menten relationship between glucose concentration and transport [13-19] was used as the basis of the proposed correction. With it, we showed that, on the average, there was less variability in untreated animals when estimating the hypothetical glucose-saturated limit to the tumor metabolic rate of glucose $\left(MR_{\mathrm{gluc}}^{\max}\right)$ rather than the tracer rate constant (*K*_i_) or the actual scan-time metabolic rate of glucose (MR_gluc_). $MR_{\mathrm{gluc}}^{\max}$ is the asymptotic limit to the plot of uptake rate versus [glucose]. *K*_M_ is a half-saturation Michaelis constant such that $MR_{\mathrm{gluc}}^{\max}=K_{i}*(K_{M}+[glc])$.

To demonstrate a true drug-induced treatment effect on glucose uptake in the tumor tissue independent of any changes in blood glucose (see Table 1 and Additional files 1 and Additional file 2), we selected dynamic FDG-PET scans from 60 mice treated with inhibitors of the cell-signaling MEK and RAF tyrosine kinases [20,21]. These have previously been reported as modulating FDG-PET in preclinical and clinical settings [22-24], and we have observed drug-induced reductions in FDG uptake both in solid tumors and in cell culture. A plausible mechanism for this reduction was demonstrated through GLUT-1 immunofluorescence. We analyzed data before and after 7 days of treatment, a compromise between early read-out and being certain that the treatment had had time to take effect.

**Table 1:** Treatment studies, cell lines, and drug substances

**Study**	**Cell line**	**Tissue Type**	**Drug substance**	**Mice**
A	A375	Melanoma	GDC-0879 (BRAF)	18
B	A2058	Melanoma	G-00033054 (MEK)	18
C	HCT116	Colorectal	GDC-0973 (MEK)	24
			TOTAL	60

Because limited experimental studies alone were inadequate to explore with any certainty the power relationships in (relatively noisy) FDG-PET data, we have supplemented these experiments with statistical simulations and with analytical derivations that are presented in Additional file 3.

## Methods

### The experimental setting

Our laboratory experiments employed dynamic FDG-PET to measure the tumor uptake rate constant for FDG, *K*_i_, as a function of tumor treatment with tyrosine kinase inhibitor drugs. The experiments contained two or more groups of animals: one control group administered vehicle alone, and at least one treatment group administered an active drug in the same dosing vehicle. We analyzed data before and after 7 days of treatment, expecting that there would be no difference between the groups before treatment and that some treatment effect would be evident after 7 days. We compared *K*_i_ with two alternative PET metrics that account for blood glucose in some way, MR_gluc_ and $MR_{\mathrm{gluc}}^{\max}$, to study the relative merits of each metric at detecting a true tumor treatment effect as seen in the two-sample two-sided *t*-test. This is also the scenario the simulations (below) and power calculations ( Additional file 3) are designed to represent.

### False positives

We considered that a true treatment effect altering tumor glucose uptake was one based on a physiological change in the tumor tissue per se. Thus, for our purposes, changes in tumor glucose uptake caused merely by alterations in blood glucose were not true treatment effects but fall into our definition of false positive results.

### Laboratory experiments

#### Animal handling and imaging

Experimental details were as described previously [14,25]. All animals were fasted overnight with access to water *ad libitum*. Mice were induced and maintained under light anesthesia using isoflurane in air (GDC-0879 study) or sevoflurane in air (G00033054 and GDC-0973 studies). Body temperature was maintained at 37°C with warm air flows while the eyes were protected from dehydration with ophthalmic ointment. All studies were conducted under the approval of Genentech's AAALAC-accredited Institutional Animal Care and Use Committee. All animals underwent 30-min dynamic FDG-PET scans with X-ray computed tomography (CT)-based attenuation correction just prior to starting their treatment regimen. FDG doses were infused via the lateral tail vein over a 1-min period in a volume of 100 μL.

#### Blood glucose measurements

At every scan, blood glucose measurements were taken twice: once approximately 5 min before and once shortly after the PET/CT scan approximately 35 min later. The glucose value used in subsequent calculations is the mean of the pre- and post-scan measurements. Data were collected with the commercially available Contour glucometer (Bayer Healthcare, Tarrytown, NY, USA) using blood freshly obtained by pricking the saphenous vein. Test-retest reproducibility measurements using this instrument in our hands showed a coefficient of variation of 3.7% [14].

#### Prior use of the experimental data

The 665 mice in 66 studies (Table 2) used here to inform the simulation parameters are mostly the same as those 585 mice described in our analysis of variability [14], refined slightly by adding in data from newly available cohorts of A375, HCT116, and MEL-537 mice and removing a small number of animals for which post-treatment scans were unavailable (H596, A2058).

**Table 2:** Animal models and number of mice

**Model**	**Cell line/strain**	**Number of cohorts**		**Number of mice**	
		**Control**	**Treatment**	**Control**	**Treated**
1	BT474 in SCID Nude Beige	2	2	22	22
2	HCT116 in Nu/Nu	5	8	54	86
3	PC3 in Nu/Nu	2	2	24	24
4	FaDu in CB17 SCID	1	1	10	10
5	H292 in CB17 SCID	1	1	10	10
6	H596 in huHGF transgenic	1	3	11	33
7	537-Mel in Nu/Nu	2	4	17	31
8	A2058 in Nu/Nu	4	10	39	99
9	A375 in Nu/Nu	4	7	35	64
10	Colo205 in Nu/Nu	1	1	12	12
11	H2122 in Nu/Nu	1	3	10	30
	Subtotal	24	42	244	421
	Total	66		665	

#### Tumor treatment models with established drug effects on tumor glucose uptake

Table 1 describes the subset of studies from Table 2 in which there was additional non-imaging evidence of a true treatment effect on tumor glucose uptake independent of blood glucose levels. Athymic nude mice were implanted in the right flank with a Matrigel/Hanks Balanced Salts medium containing 10 million melanoma (A375, A2058) or 5 million colorectal (HCT116) cancer cells. Tumors reached a group median volume of at least 250 mm^3^ prior to beginning the study. The blood glucose and FDG-PET data (*K*_i_, MR_gluc_, $MR_{\mathrm{gluc}}^{\max}$) are presented for these studies in Additional file 1. Cell culture experiments were used to show direct drug effects on FDG uptake, and immunofluorescence was used to show an apparent loss of GLUT-1 at the cell membrane both in cells and tumor tissue (see Additional file 2 for descriptions of and results for those experiments).

#### Statistical power in experimental data: p-values as a function of sample size

Two-sample two-sided *t*-test *p*-values were calculated in these three true treatment studies: A, B, and C described in Table 1. This was repeated using, $MR_{\mathrm{gluc}}^{\max}$, MR_gluc_, and *K*_i_. We examined the *p*-values at baseline, where the null hypothesis should be accepted, and on treatment at day 7, where the null hypothesis should indeed be rejected based on our knowledge of drug action on tumor cell and tissue glucose handling ( Additional file 2).

**Figure 1:**
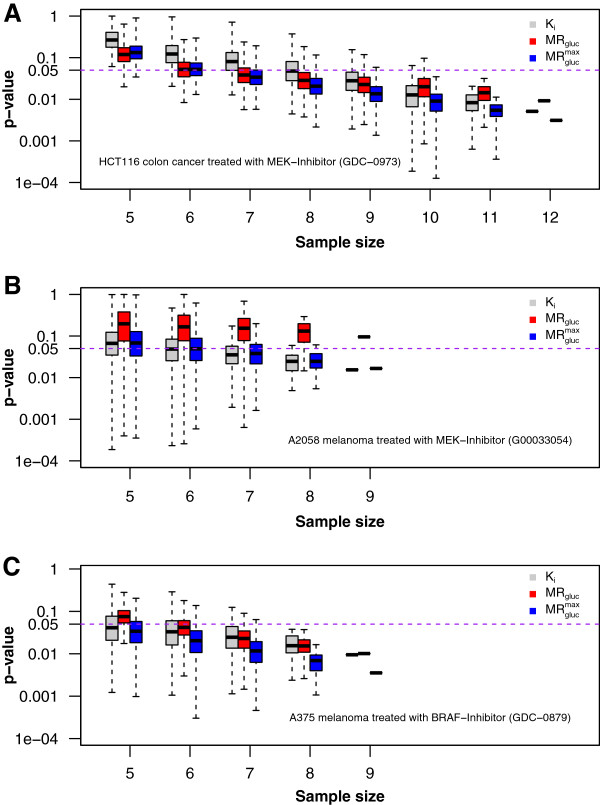
**Experimental statistical power at day 7 post-dose.** Three panels correspond to three animal models from Table 2. Each shows Student's *t*-test results from treatment comparisons of control and treatment groups of mice as a function of sample size and using three PET metrics. (**A**) HCT116 colorectal cancer in Nu/Nu mice. (**B**) A2058 melanoma cancer in Nu/Nu mice; (**C**) A375 melanoma cancer in Nu/Nu mice. Results were calculated for the full group size of *n* animals and for all possible combinations of individuals (limited to a maximum of 4,000 random samples) studied in four progressively smaller subsets (*x*-axis). The *y*-axis (log_10_ scale) indicates the significance level *p*-value. The purple dashed line indicates a significance level of 0.05. Every boxplot includes a bold horizontal line that indicates the median *p*-value. The box length shows the interquartile range (25% to 75%), and the whiskers show minimum and maximum observed *p*-values.

A preliminary analysis confirmed that our A375 (*n* = 9), A2058 (*n* = 9), and HCT116 (*n* = 12 per group) tumor studies were powered with sufficient numbers of animals to detect large treatment effect sizes using any FDG-PET metric: *K*_i_, MR_gluc_, or $MR_{\mathrm{gluc}}^{\max}$. To examine how studies with less power might perform, we undertook the simulations described below and supplemented those with a meta-analysis of smaller groups obtained by sampling within our experimental data. We considered the full cohort of animals prepared for a given study to be the ‘universe’ of animals from which the smaller groups were drawn randomly using sampling without replacement. We calculated results (presented in Figure 1) for every possible combination of individuals as long as the number of combinations totaled less than 4,000; when more combinations were possible, we randomly sampled 4,000 cases to generate our results.

#### False positive rates in experimental data: relation to sample size

Mice were randomized into nominal control and treatment groups, each containing *n* = 6 to 12 mice (Table 2), allowing 42 comparisons of two-sample two-sided *t*-tests to be performed on FDG-PET data collected before any treatment was administered. At this timepoint, a statistically significant result was considered to represent a false positive result. A particular study was flagged as having a high rate of false positives whenever the *t*-tests rejected the null hypothesis (*p* < 0.05) more often than the theoretical false positive rate (α) of 5%, measured across all the combinations of individuals tested. Meta-analysis of progressively smaller subsets as described above was used to assess how the false positive error rate would behave in smaller, less powerful, studies. This was repeated using $MR_{\mathrm{gluc}}^{\max}$, MR_gluc_, and *K*_i_.

#### Pharmaceuticals

GDC-0879 is a B-RAF [20] selective kinase inhibitor [26,27] that has been demonstrated to be effective against cancers carrying the V600 mutation [28]. MEK is one of the three enzymes of the mitogen-activated protein kinase (MAPK) cascade involved with RAS/RAF signaling [21]. G00033054 and GDC-0973 are potent and selective MEK inhibitors that have been efficacious in treating KRAS and RAF mutant cells [29].

All drug substances were dosed daily in 100 μL of excipient. GDC-0879, GDC-0973, and G00033054 were dosed for 7 days at 100 mg/kg, 10 mg/kg, and 25 mg/kg, respectively. All animals were dosed through oral gavage (*per os*). Control groups were subjected to the same regimen but received no active drug in their dosing solution.

### Derivations, statistics, and simulations

We studied the properties of the two-sample two-sided *t*-test comparing sample means of *K*_i_ and $MR_{\mathrm{gluc}}^{\max}$ between control and treatment groups, respectively, in analytical derivations (presented as Additional file 3) and in simulations which are described below. Data were simulated assuming either no treatment effect or assuming a treatment effect of 10% to 50% change in the glucose-saturated limit to the tumor glucose uptake rate, $MR_{\mathrm{gluc}}^{\max}$, specified in each simulation. As a function of the involved parameters, our study evaluated the test statistics under both the null and alternative hypotheses by estimation of false positives (including significant test results caused merely by changes in blood glucose) and the power to detect true differences in the tumor glucose uptake rate limit. Simulations were run in the statistical programming language R [30].

We assumed that the relationship between the FDG rate constant *K*_i_ and glucose [glc] followed the Michaelis-Menten (MM) form [14-19] and that observations of the rate constant were corrupted by noise. That is, the observed rate constant was given by $K_{i}=MR_{\mathrm{gluc}}^{\max}/(K_{M}+[glc])+\varepsilon$, where *ε* is the zero-mean Gaussian with variance $\sigma_{\varepsilon }^{2}$, here denoted as $\varepsilon ∼N(0,\sigma_{\varepsilon }^{2})$. Let ${\bar{K}}_{\text{i}}^{\text{C}},{\bar{K}}_{\text{i}}^{\text{T}}$ represent the sample average FDG uptake rates across *n* observations in the control and treatment groups, respectively, and let ${\bar{\text{MR}}}_{\text{gluc}}^{\text{max,C}}$ and ${\bar{\text{MR}}}_{\text{gluc}}^{\text{max,T}}$ be the sample averages of the quantity *K*_i_ * (*K*_M_ + [glc]) in the two groups. Under these assumptions, we compared the statistical properties of the *t*-test comparing ${\bar{K}}_{\text{i}}^{\text{C}}$ and ${\bar{K}}_{\text{i}}^{\text{T}}$ with the *t*-test comparing ${\bar{\text{MR}}}_{\text{gluc}}^{\text{max,C}}$ and ${\bar{\text{MR}}}_{\text{gluc}}^{\text{max,T}}$.

The analytical derivation of the power functions relating to *K*_i_ and $MR_{\mathrm{gluc}}^{\max}$ follows standard developments based on the Gaussian distribution [31] and is presented for the interested reader in Additional file 3. To illustrate the validity of the derivation and to delineate when $MR_{\mathrm{gluc}}^{\max}$ provides significantly improved statistical properties *vis-à-vis K*_i_, we simulated observations from the joint process (*K*_i_, [glc]) as follows. Given the parameters $\left\{MR_{\mathrm{gluc}}^{\max},K_{M},\mu_{g},\sigma_{g}^{2},\sigma_{\varepsilon }^{2}\right\}$, a single draw of (*K*_i_, [glc]) was obtained by first sampling $\left[glc]∼N(\mu_{g},\sigma_{g}^{2}\right)$ and $\varepsilon ∼N(0,\sigma_{\varepsilon }^{2})$, and then by evaluating $K_{i}=MR_{\mathrm{gluc}}^{\max}/(K_{M}+[glc])+\varepsilon$. For each simulation iteration, the preceding was repeated *n* times each in the control and treatment groups, respectively, and two-sided *t*-tests were used to test for equality of means at *α* = 0.05 level of significance. A total of 4,000 simulation iterations were used in each setting.

**Figure 2:**
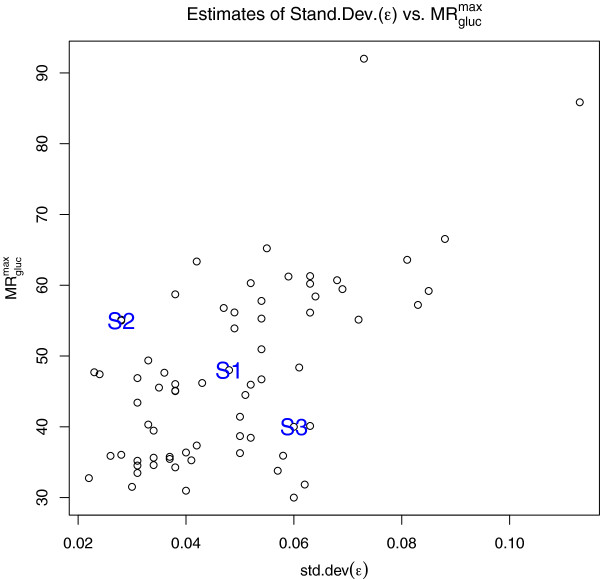
**Estimates of**${\text{MR}}_{\text{gluc}}^{\text{max}}$**and standard deviation (*ε*) in the 66 studies described in Table**2**.** Illustrative cases discussed in the text are marked as S1, S2, and S3.

To get representative simulations, we chose parameter values based on output from fitting the MM model to FDG-PET data from each of the 66 (as-yet-untreated) experimental cohorts of mice described in Table 2. For these studies, with the half-rate Michaelis constant set at *K*_M_ = 130 mg/dL [14], the scatter plot in Figure 2 shows estimates of $MR_{\mathrm{gluc}}^{\max}$ versus σ_ε_. For $MR_{\mathrm{gluc}}^{\max}$, the sample mean and standard deviation were 47.9 and 12.7, respectively (range = 31.0 to 92.0), and for σ_ε_, they were 0.048 and 0.018, respectively (range = 0.022 to 0.113). Based on these values, the first simulation setting (‘S1’, noted on the face of Figure 2) represents an ‘average’ case with $MR_{\mathrm{gluc}}^{\max}$ and σ_ε_ set at their sample mean values of 48 and 0.048. The second (‘S2’) and third (‘S3’) settings (likewise noted on the face of Figure 2) represent cases with strong and weak signal-to-noise ratios, where $MR_{\mathrm{gluc}}^{\max}$ and σ_ε_ are set to (55, 0.028) and (38, 0.057), respectively. In each simulation, glucose was sampled according to [glc] ~ *N*(90, 25^2^), the approximate marginal distribution of glucose across the sample data, and *K*_M_ remained fixed at 130 mg/dL.

For simulations under the null hypothesis, the maximal uptake rate $MR_{\mathrm{gluc}}^{\max}$ was set the same in the control and treatment groups, and we evaluated the effect on the false positive rate (i.e., concluding that there is a treatment effect when in fact there is none) caused merely by a change in mean blood glucose. Mean blood glucose changes of 10%, 20%, and 30% were assessed.

Simulations under the alternative hypothesis compared the power of the *t*-tests to detect treatment effects (*δ*) corresponding to an approximate 20% to 30% reduction in the tumor glucose uptake rate limit $MR_{\mathrm{gluc}}^{\max}$ while keeping the glucose distribution the same. Sample sizes were chosen between *n =* 6 and *n =* 12.

The robustness of $MR_{\mathrm{gluc}}^{\max}$ to errors in [glucose] and *K*_M_ was also investigated by simulations. For errors in the measurement of blood glucose, we replaced the quantity *K*_i_ (*K*_M_ + [glc]) by *K*_i_ (*K*_M_ + [glc]*), where [glc]* = [glc] + *N*(0, 4^2^). That is, the *K*_i_ values were generated using the correct (uncorrupted) glucose values [glc], while $MR_{\mathrm{gluc}}^{\max}$ was estimated using observed (corrupted) glucose [glc]*. A similar process of substitution was used with *K*_M,_ using scenarios (*K*_M_ = 100 mg/dL, *K*_M_* = 130 mg/dL) and (*K*_M_ = 130 mg/dL, *K*_M_* = 100 mg/dL).

## Results and discussion

### Results

#### Statistical and blood glucose-induced false-positive error rates

In the absence of any glucose bias between the control and treatment groups, the *t*-tests based on *K*_i_, $MR_{\mathrm{gluc}}^{\max}$, and MR_gluc_ all have simulated false positive rates which are consistent with the nominal statistical type I false positive error rate of *α* = 0.05. However, as seen in Table 3, for the first simulation setting with *n* = 12 observations per group, only the test based on $MR_{\mathrm{gluc}}^{\max}$ preserves the correct false positive error rate in the presence of a glucose bias, while the tests based on *K*_i_ and MR_gluc_ both perform increasingly poorly as the magnitude of the bias grows. The increase in the false positive rate can be understood by noting that any glucose bias induces a shift in *K*_i_ that is false with regard to effects intrinsic to the tumor. Specifically, under the Michaelis-Menten model, a shift in mean glucose between the control and treatment groups by *δ*_g_ units translates into an approximate (first-order) $-MR_{\text{gluc}}^{\text{max}}/{\left(K_{M}+\mu_{g}\right)}^{2}\times \delta_{g}$ change in the mean level of *K*_i_ (see Additional file 3). For instance, in the first simulation setting S1, a 30% average increase in mean glucose from *μ*_g_ = 90 in the control to 117 mg/dL in the treatment group induces a false, average change in *K*_i_ of −0.0268 per second or approximately −11.0%. Substituting for *δK*_i_ in the analytical power equation (see Equation 1 in Additional file 3) yields an estimated false positive error rate of 19.3%, in close agreement with the simulated value of 18.4% (see Table 3). The same strong effect on the false positive error rate due to a glucose shift was observed for the second and the third simulation settings, S2 and S3 (results not shown).

**Table 3:** False positive error rates (%)

**Glucose bias**	**−30%**	**−20%**	**−10%**	**0%**	**10%**	**20%**	**30%**
*K*_i_	25.7	13.1	6.5	4.8	6.5	12.5	18.4
$MR_{\mathrm{gluc}}^{\max}$	5.0	4.9	5.4	4.9	4.9	5.0	4.5
MR_gluc_	41.8	18.2	8.6	5.1	6.8	14.2	23.6

The error rates are expressed as percentages for a two-sided *t*-test at level *α* = 0.05 based on $K_{i},MR_{\mathrm{gluc}}^{\max}$, and MR_gluc_ as a function of glucose bias. Glucose bias is defined as the percent change in mean glucose between the control and treatment groups. Here, $MR_{\mathrm{gluc}}^{\max}=48,\sigma_{\varepsilon }=0.048,n=12$.

As predicted by the derivations, all three metrics (*K*_i_, MR_gluc_, and $MR_{\mathrm{gluc}}^{\max}$) correctly accepted the null hypothesis at baseline in the 42 comparisons of the control with treatment groups in the full experimental data (Table 2). Also as expected, false-positive results began to appear as the data were resampled at smaller sample sizes. At sample size *n* = 8, for example, only one comparison showed high false positive rates by *K*_i_ and $MR_{\mathrm{gluc}}^{\max}$, at which point MR_gluc_ gave false positives in 6 out of the 42 studies (14%).

#### Elimination of MR_gluc_ from further consideration

Because results based on MR_gluc_ were highly influenced by relatively modest levels of glucose bias (Table 3), results that we considered to be false in terms of treatment response, we judged that the most suitable alternative to $MR_{\mathrm{gluc}}^{\max}$ was the (uncorrected) *K*_i_. We henceforth simplify the presentation of simulation results and analytical derivations by restricting them only to *K*_i_ and $MR_{\mathrm{gluc}}^{\max}$. The performance of MR_gluc_ in the experimental data is, however, shown alongside *K*_i_ and $MR_{\mathrm{gluc}}^{\max}$ ( Additional file 1 and Figure 1).

#### Statistical power in theory and in simulation

As shown in the analytical power derivations presented in Additional file 3, an improvement in power for $MR_{\mathrm{gluc}}^{\max}$, *P*_*m*_, relative to the power for *K*_i_, *P*_*k*_, occurs whenever the coefficient of variation (CV) in *K*_i_ evaluated at the mean glucose level is less than 1. That is, with *P*_*k*_, *P*_*m*_ the power curves for a test of means of *K*_i_ and $MR_{\mathrm{gluc}}^{\max}$, respectively, then, whenever CV = *σ*_*ε*_/*K*_i_(*μ*_g_) < 1, where $K_{i}(\mu_{g})=MR_{\mathrm{gluc}}^{\max}/(K_{M}+\mu_{g})$, we have *P*_*m*_ >*P*_*k*_. Moreover, through manipulation of Equations 1 and 2 in Additional file 3, we see that the difference *P*_*m*_ − *P*_*k*_ is monotonic, increasing with decreasing CV. Further, the difference *P*_*m*_ − *P*_*k*_ grows as $\sigma_{g}^{2}$ increases (holding CV constant). We now detail these facts by simulation.

**Figure 3:**
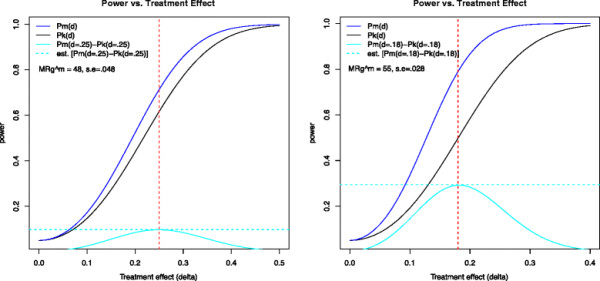
**Power curves as a function of the treatment effect (*δ*).** Simulation settings S1 and S2 are as shown in Figure 2. In S1 (left), $MR_{\mathrm{gluc}}^{\max}=48,\sigma_{\varepsilon }=0.048,n=10$, and in S2 (right), $MR_{\mathrm{gluc}}^{\max}=55,\sigma_{\varepsilon }=0.028,n=6$. The solid blue and black lines represent the theoretical power curves for $MR_{\mathrm{gluc}}^{\max}$ and *K*_i_, respectively (see derivations in Additional file 3), while the solid cyan lines show the power improvement. The dotted cyan line shows the peak simulated improvement in power for the two settings S1 and S2 at *δ* = 0.25 and *δ* = 0.18, respectively.

Figure 3 shows the theoretical power curves *P*_*k*_ (blue solid line) and *P*_*m*_ (black) for the first and second simulation settings, S1 (left panel) and S2 (right panel). The first case, S1, represents an average study with parameters $MR_{\mathrm{gluc}}^{\max}$ and σ_ε_ set at the mean levels and with *n =* 10; a potential improvement of approximately 10% occurs at a treatment effect of *δ* = 0.25 (cyan solid line), with a corresponding simulated improvement of 9.8%. The second case, S2, exemplifies a study with a particularly good signal-to-noise ratio, i.e., low σ_ε_. Here, an improvement of approximately 29.2% occurs for *δ* = 0.18, with a simulated improvement of 29.9%.

The third simulation case, S3, representing very noisy data where $MR_{\mathrm{gluc}}^{\max}=38,\sigma_{\varepsilon }=0.057,n=10$, has a maximum improvement in power of 2.2%, occurring for *δ* = 0.55 (plot not shown). This indicates that with low signal-to-noise ratios in the *K*_i_ measurement, there is no meaningful improvement in power from using $MR_{\mathrm{gluc}}^{\max}$. However, cases with high coefficient of variation inevitably have low power and require either very large treatment effects or very large sample sizes to detect a difference in means. Indeed, for case S3, we would require *n* = 40 for 80% power to detect a treatment effect of *δ* = 0.25.

**Figure 4:**
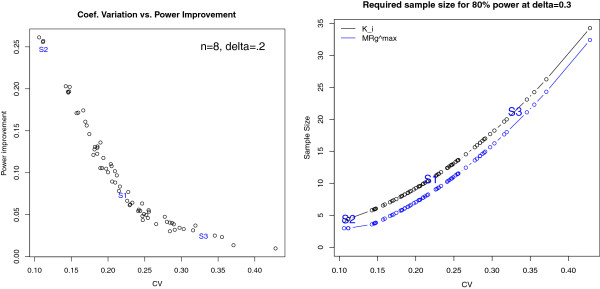
**Power improvement.** Left panel: power improvement in using $MR_{\mathrm{gluc}}^{\max}$ compared to *K*_i_ when *n* = 8, *δ* = 0.2 as a function of the CV in *K*_i_ for the 66 studies of Table 2. Right panel: scatter plot estimates of the sample size required to reach 80% statistical power as a function of the CV in *K*_i_. The power curve for *K*_i_ is shown in black and for $MR_{\mathrm{gluc}}^{\max}$, in blue. The parameter values and power improvement of the three simulation settings S1, S2, and S3 are depicted for reference in each plot.

For the case *n* = 8, *δ* = 0.3, the left panel of Figure 4 shows the power improvement as a function of the coefficient of variation across the 66 cohorts considered (Table 2). The right panel of Figure 4 offers an alternative perspective on this power improvement, being the sample size required to perform a well-powered study (80% chance of correctly rejecting the null hypothesis). An average study that requires 10 animals per group using *K*_i_ is equivalently powered using 8 animals per group with $MR_{\mathrm{gluc}}^{\max}$. In addition, the $MR_{\mathrm{gluc}}^{\max}$ measurements resist false positive results in the event of glucose bias.

Congruent with the main result outlined in the derivations presented in Additional file 3, the improvement in power is strongly dependent on the coefficient of variation in *K*_i_, with the largest power improvement reaching approximately 25%. Moreover, the greater the coefficient of variation for *K*_i_, the less we can discern the effects due to glucose; however, as noted, no test performs well with excessively noisy data.

#### Statistical power in experimental data

On the average and in agreement with the simulations, $MR_{\mathrm{gluc}}^{\max}$ gave greater power than *K*_i_ or MR_gluc_ in detecting the known direct on-tumor drug effects in all three tumor treatment models studied (Table 1 and Figure 1). As expected, all metrics progressively lost power as the sample size decreased. For example, in Figure 1A at eight mice per group, $MR_{\mathrm{gluc}}^{\max}$ was able to reject the null hypothesis in 93% of the 4,000 combinations of control vs. treatment groups, while *K*_i_ did so in only 52% of the sample combinations. In Figure 1B, MR_gluc_ completely misses the treatment effect at all sample sizes, but *K*_i_ and $MR_{\mathrm{gluc}}^{\max}$ correctly identified it. Lastly, in Figure 1C, looking at six mice per group, we observe that $MR_{\mathrm{gluc}}^{\max}$ detected a statistically significant difference between the groups, 89% of all the sample combinations, while MR_gluc_ did so in only 62% of the cases. However, caution must be exercised in drawing fully general conclusions from these limited and somewhat noisy experimental data alone.

### Discussion

#### Application of MR_glu*c*_

The original intent behind the multiplication of *K*_i_ by [glucose] was to estimate the metabolic rate of glucose (MR_gluc_) in tissue under given blood glucose levels based on rate constants derived from monitoring a radioactive glucose-like tracer in blood and tissue [13,32]. The estimation implies the assumption that MR_gluc_ depends on substrate concentration, i.e., [glucose] in blood. It follows that MR_gluc_ is unsuitable for our particular task of quantitatively compensating for changing glucose levels when comparing scans collected under different glucose conditions. Our results show that even seemingly small differences in blood glucose, such as the natural variations within a group of similar individuals, are sufficient to warrant careful attention to glucose correction when making quantitative comparisons.

#### The lumped constant

Measurement of the lumped constant (LC) is not trivial, and thus, the (ideal) per-patient or per-lesion values are rarely measured and reported with FDG-PET treatment studies. Instead, a common constant value of LC is applied to all scans. This approach was employed in this study too with an assumed LC value of 1, and as previously noted [14], the chosen value of LC simply behaves as a scaling factor common to every data point and thus makes no difference to calculated group statistics such as the coefficient of variation, *t*-test *p*-values, or correlations with blood glucose levels. The statistical results presented remain equally valid at all (non-zero) values of LC.

#### Glucose bias and false positive test results

All three metrics performed correctly in terms of the false positive rate in the absence of any systematic glucose difference between the treatment groups. The fact that the *t*-tests based on *K*_i_ and MR_gluc_ suffer an increased false positive error rate under a glucose shift (Table 3) renders these tests admissible and useful only if one is certain that a treatment can have no systematic effect on glucose. Since blood glucose levels may vary, we suggest that $MR_{\mathrm{gluc}}^{\max}$ makes a more robust and useful default metric for FDG-PET data.

#### Statistical power in the absence of any glucose bias

Figure 4 (left hand side) shows the simulated improvement in power for a modest treatment effect of 20% and a sample size of *n* = 8. As can be seen, the power improvement can be as large as 25% and is highly dependent on CV. As predicted by the analytical derivations, the benefit of using $MR_{\mathrm{gluc}}^{\max}$ is most pronounced at low CV. Conversely, for values of CV greater than 35%, the power benefit is negligible even though the benefit of reduced glucose bias remains. However, for data that is very variable (relative to the mean), larger treatment effects or sample sizes are always required for adequate power, a fact that is detailed in the right hand plot of Figure 4.

Figure 4 (right hand side) shows the required sample size for *K*_i_ and $MR_{\mathrm{gluc}}^{\max}$ as a function of the coefficient of variation in order for a study to have 80% power with a treatment effect size of 30% (*δ* = 0.3). As expected, for both *K*_i_ and $MR_{\mathrm{gluc}}^{\max}$, the required sample size is an increasing function of the CV value. We see that a CV of 22% (the average in our experiments) requires a sample size of *n* = 10 per group for *K*_i_ and *n* = 8 per group for $MR_{\mathrm{gluc}}^{\max}$. To further describe the results, we can assume a fixed sample size and consider what proportion of our 66 experimental cohorts represented adequately powered groups for a treatment study: For the sample size of *n* = 8, we see that 48% were adequately powered using $MR_{\mathrm{gluc}}^{\max}$, whereas only 26% were adequately powered with *K*_i_. For a sample size of *n* = 12 there are more adequately powered groups, of course, but still a benefit to using $MR_{\mathrm{gluc}}^{\max}$: 76% using $MR_{\mathrm{gluc}}^{\max}$ and 59% using *K*_i_. Independent of CV, the sample size savings achieved through the use of $MR_{\mathrm{gluc}}^{\max}$ in this simulation setting is approximately two mice; in (relatively rare) situations where a CV as low as 10% can be anticipated, we see that studies can be adequately powered with only a handful of animals per group.

Understanding this behavior has practical value in designing appropriately powered preclinical FDG-PET experiments and, perhaps, in permitting a futility analysis to be conducted after beginning a study with baseline scans and before expending further significant effort in drug dosing and repeated scanning.

#### Glucose ‘normalization‘ and errors in the measurement of blood glucose

Glucose sampling errors have been postulated as a source of variability experienced [9,10] when applying the common *[glucose]/constant* normalization method [33] which is analogous to estimating MR_gluc_ at the population mean glucose measurement (the value of the constant), typically given as 5 mM or 100 mg/dL.

We suggest that the problem with this normalization scheme lies not with the glucose measurements, but with the linear nature of the algorithm. Rather than linear scaling to the population mean glucose value, $MR_{\mathrm{gluc}}^{\max}$ asymptotically follows the Michaelis-Menten extrapolation to a hypothetical saturating glucose level. Simulations showed that $MR_{\mathrm{gluc}}^{\max}$ results were robust even with relatively large 10% errors in the glucose measurements (full results not shown). This can be intuited by noticing that the *K*_M_ term is on the order of the [glucose] term, making the glucose measurement error, *ε*_glc_, a small part of the total correction factor, *K*_M_ + [glc] + *ε*_glc_. We also note that the algebraic form of this correction factor, i.e., *[glucose] + constant*, appears as a solution in analytical derivations that simply start with the very general assumption that *K*_i_ is negatively correlated with [glucose] over a limited range of glucose values. This is presented in Additional file 3 for the interested reader.

#### Optimal group comparisons with linear regression

We note that $MR_{\mathrm{gluc}}^{\max}$ is optimally estimated by regressing *K*_i_ on the quantity 1/(*K*_M_ + [glc]) under the Michaelis-Menten model assumptions specified, with the noise process *ε* following the Gaussian distribution and with a fixed value for *K*_M_. Here, we condition on the glucose measurements and set the intercept to zero. Given our setup, in the regression framework, the *t*-test of equality of the maximal uptake rates $MR_{\mathrm{gluc}}^{\max,C}$ and $MR_{\mathrm{gluc}}^{\max,T}$ is a likelihood ratio test and the uniformly most powerful unbiased test [34]. Moreover, statistically speaking, the regression estimator is best linear unbiased under non-Gaussian assumptions [35]. We also note that the variance of the regression estimator and that of the sample average ${\bar{\text{MR}}}_{\text{gluc}}^{\text{max}}$ are close provided that the spread in the term (*K*_M_ + [glc]) is low relative to its mean. In our setting, since $\sigma_{\text{g}}/\left(K_{M}+\mu_{g}\right)\approx 0.1$, the linear regression and sample average solutions are very close to each other, and either may be used when testing for a treatment effect. Thus we expect that the familiar and straightforward use of sample means (averaging data from multiple individuals) will be satisfactory when using $MR_{\mathrm{gluc}}^{\max}$ in practice, just as it is for *K*_i_.

## Conclusions

Quantitative comparisons of FDG-PET scans across time or between animals are subject to an elevated risk of erroneous results when they ignore blood glucose levels. Multiplying PET data by blood glucose levels or ‘normalizing’ the blood glucose to a common reference value (100 mg/dL, for example) offers no protection; in fact, it is frequently counterproductive. However, by calculating the hypothetical value of the maximum glucose uptake rate under saturating glucose conditions, $MR_{\mathrm{gluc}}^{\max}$, we see reduced problems of glucose bias and gain increased statistical power to detect treatment effects. Based on the average properties observed across 66 preclinical cohorts, the power improvement for $MR_{\mathrm{gluc}}^{\max}$ was equivalent to reducing the sample size by 20% compared to the next best option, which was using the uncorrected *K*_i_ data.

These benefits were realized in our preclinical studies of tyrosine kinase inhibitors by computing $MR_{\mathrm{gluc}}^{\max}=K_{i}*\left(K_{M}+[glc]\right)$ using a *K*_M_ of 130 mg/dL. The analytical derivations and simulation methods described in this work should facilitate the exploration and assessment of our method in other settings. Because it is superior to making no glucose correction and its benefits are easily obtained and come with no penalty, we highly recommend the use of (*K*_M_ + [glc]) rather than [glucose] or [glucose]/(100 mg/dL) as the glucose correction factor in quantitative FDG-PET studies.

## Competing interests

The authors declare that they have no competing interests.

## Authors’ contributions

SPW initiated and directed the work and wrote the manuscript. JEF helped with the PET experiments and image analysis, and undertook the data curation and mining, statistical analysis, and figure preparation, AB undertook the treatment validation work presented in Additional file 2. RP critically reviewed the data analysis, figures, and manuscript. TB oversaw the statistical analysis, ran the simulations, and prepared the mathematical analysis of Additional file 3. All authors read and approved the final manuscript.

## Supplementary Material

Additional file 1 **Title: FDG-PET data *in vivo *for the three true treatment studies. ** Description: Summary results for tumor FDG-PET in three exemplary xenograft models. Click here for file

Additional file 2 **Title: FDG *in vitro *and GLUT-1. ** Description: Evidence of drug action on cellular glucose uptake *in vitro * and *in vivo * for the same three models presented in Additional file 1. Click here for file

Additional file 3 **Title: Analytical derivations of the power functions. ** Description: Statistical power calculations: analytical derivation. Click here for file
