# Supplementary material for: The power of FDG-PET to detect treatment effects is increased by glucose correction using a Michaelis constant
Source: EJNMMI Res. 2012 Jun 27;2:35. doi: 10.1186/2191-219X-2-35 (PMC3472222; doi:10.1186/2191-219X-2-35)
Supplement: Additional file 1 — Title: FDG-PET data in vivo for the three true treatment studies. Description: Summary results for tumor FDG-PET in three exemplary xenograft models. [file 2191-219X-2-35-S1.pdf]

Additional File 1 – FDG PET data in vivo for the three true treatment studies

FDG-PET response of HCT116 colorectal tumors treated with GDC-0973 (10 mg/kg, P.O. daily)

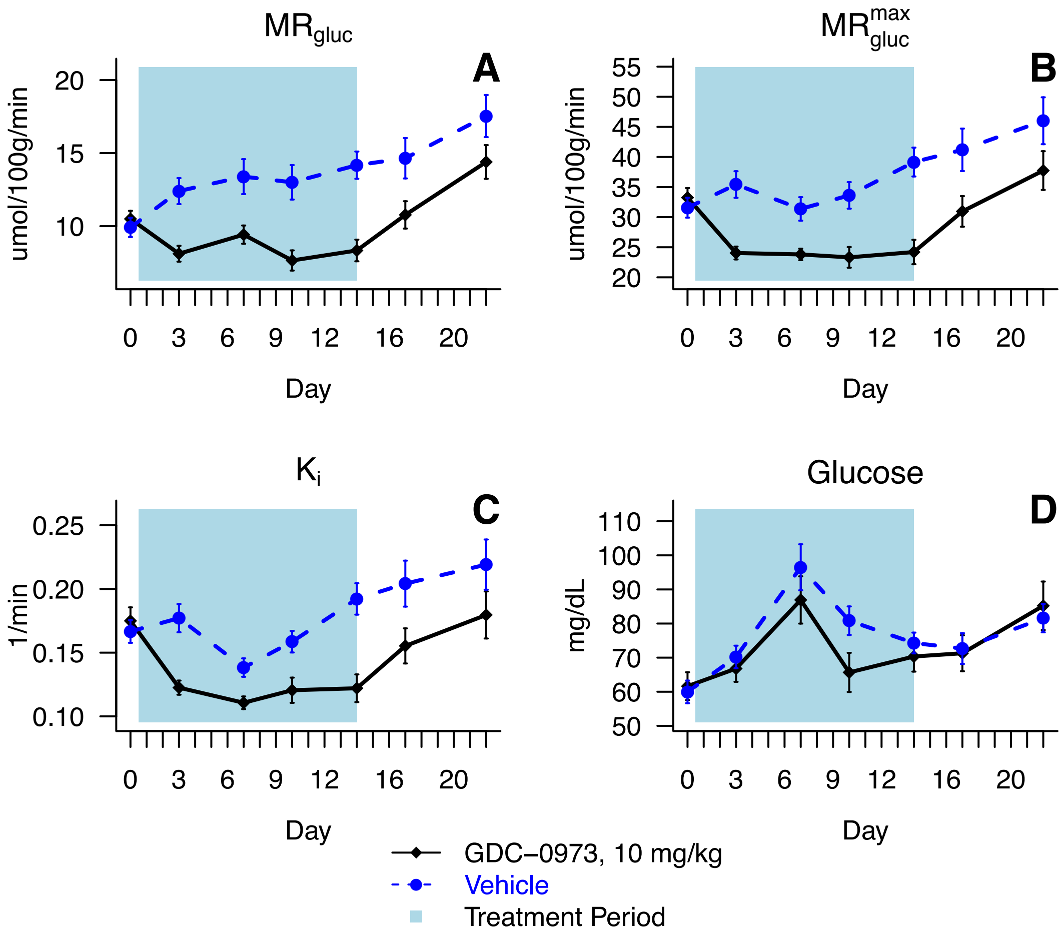

Note: Normal fasting procedure is to move the animals into a clean cage with no food. The day 7 spike in glucose levels was caused by erroneously leaving the animals in their old cages while simply removing the food basket – evidently this is less effective.

FDG-PET response of A2058 melanoma treated with G00033054 (25 mg/kg, P.O. daily)

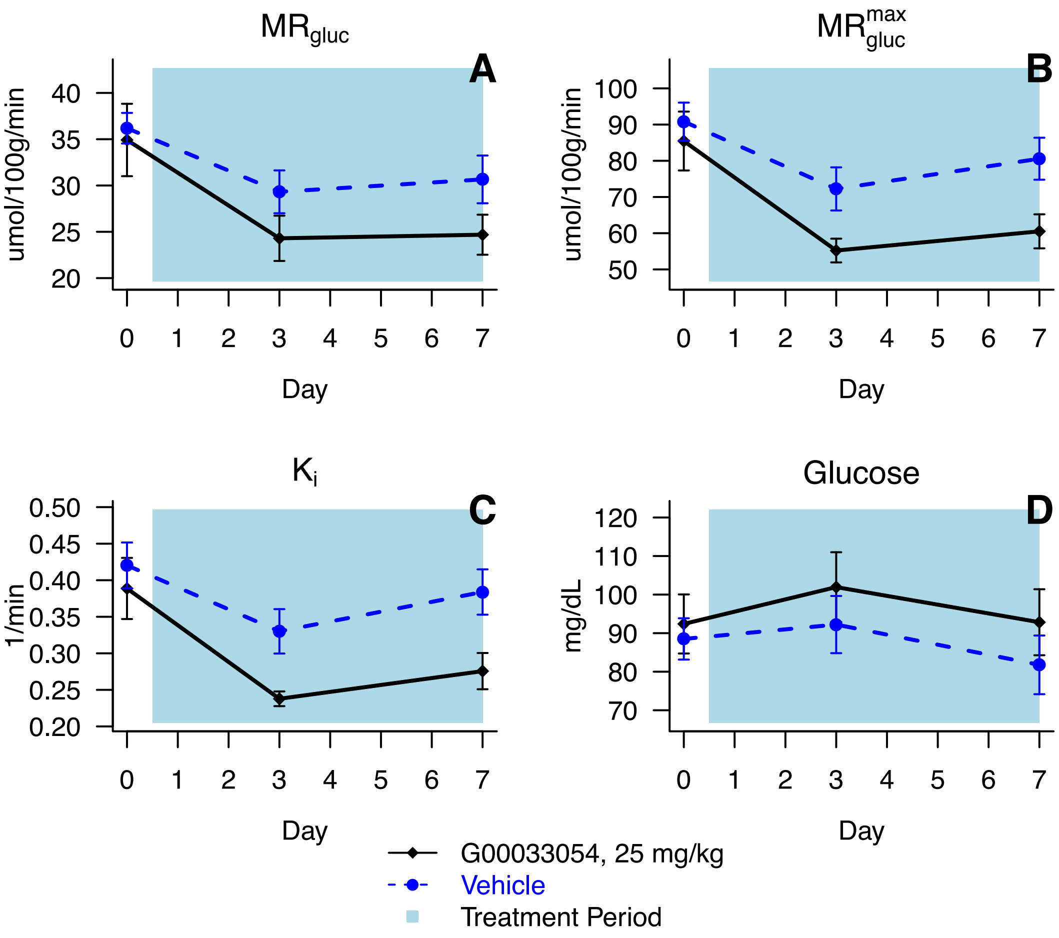

Longitudinal study of A2058 melanoma tumors in 9 mice per group studies over 7 days. The panels show the behavior of MR_{gluc} (A), MR_{gluc}^{max} (B), K_i (C), and blood glucose (D).

FDG-PET response of A375 melanoma tumors treated with GDC-0879 (100 mg/kg, P.O. daily)

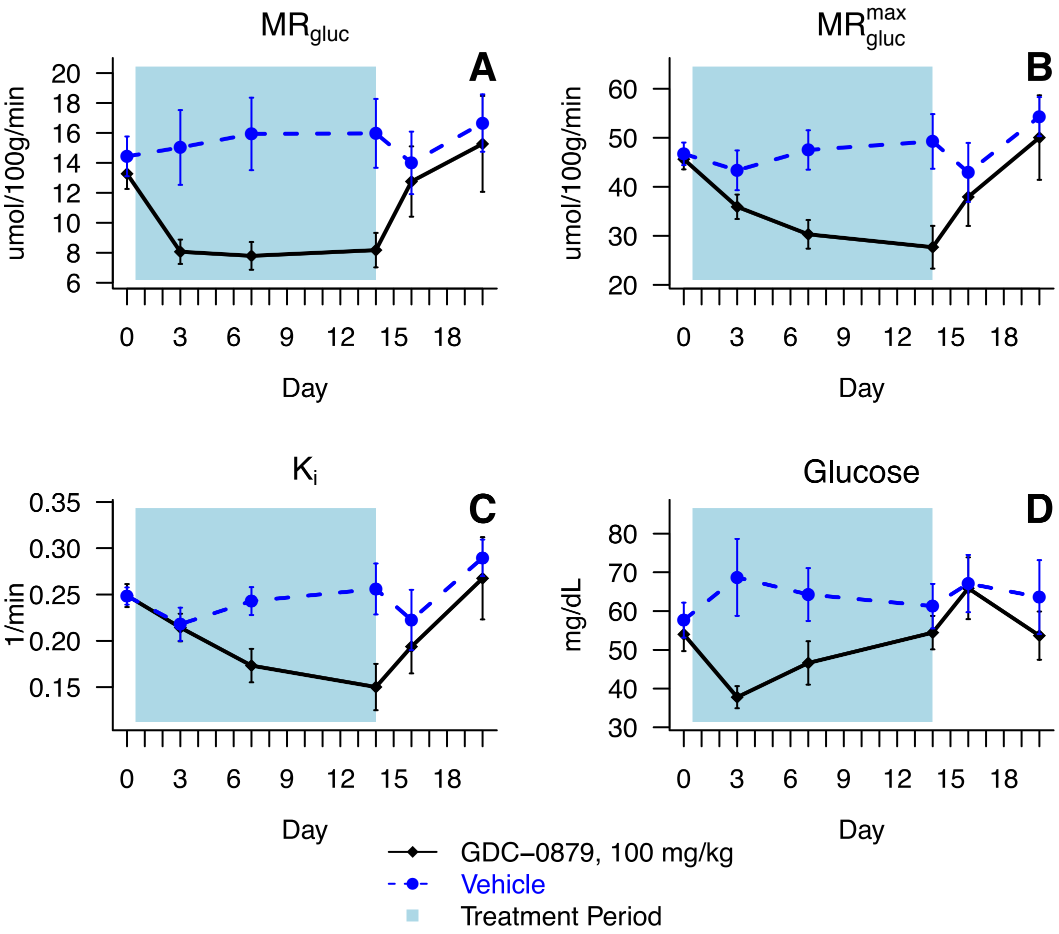

Note: On day 3, treatment groups showed very different glucose numbers between each other. Since the systematic glucose bias is present in both treatment groups in opposite directions, MR_{gluc} detected an apparent treatment effect, while K_i does not detect any difference at all.
