# Supplementary material for: The power of FDG-PET to detect treatment effects is increased by glucose correction using a Michaelis constant
Source: EJNMMI Res. 2012 Jun 27;2:35. doi: 10.1186/2191-219X-2-35 (PMC3472222; doi:10.1186/2191-219X-2-35)

Additional File 2 –
FDG in vitro and GLUT-1

FDG uptake in-vitro

METHODS

On Day 0, cells were plated out in 96-well Cytostar-T scintillating microplates at a concentration of 25,000 cells/well in the recommended culture medium (DMEM supplemented with 10% FBS, 2mM L-Glutamine). On Day 1, drug substances dissolved in DMSO were spiked into the medium, and on Day 2 the medium was aspirated from the plates and the cells washed once with Krebs-Ringer buffer. Cells were covered with "low glucose" DMEM (100mg/dL D-glucose) containing 2.7µCi of 2-^{18}F-fluoro-2-deoxy-D-glucose ("FDG") and 5µM Vybrant DyeCycle Ruby vital nuclear stain and allowed to incubate for 1hr while maintained at 37 °C. Plates were then washed 5 times with chilled Krebs-Ringer buffer, fixed in 4% paraformaldehyde, and the radioactivity measured using a Microbeta^2 Plate counter (Perkin-Elmer).

RESULTS

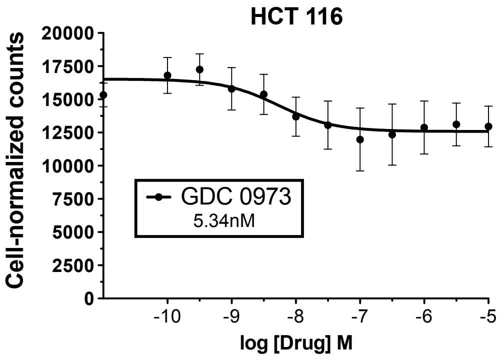

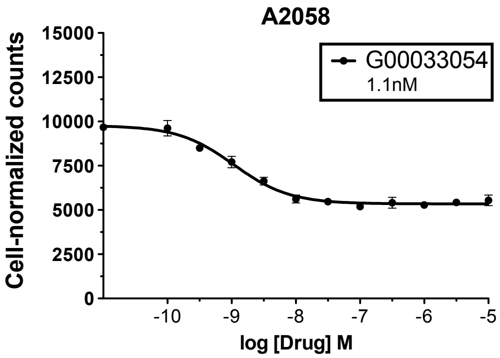

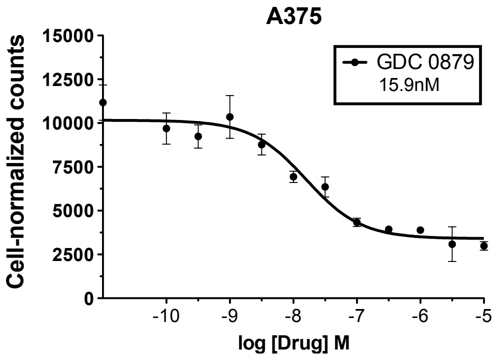

GLUT-1 immunofluorescence in vitro

METHODS

Following drug treatments as described above for the FDG uptake assays, GLUT-1 immunofluorescence assays were performed with an anti-GLUT-1 primary antibody (Chemicon #AB1340) and an Alexa-488 secondary antibody (Invitrogen #A11008) on formaldehyde-fixed, Triton-permeabilized cells blocked with 5% fish gelatin.

RESULTS

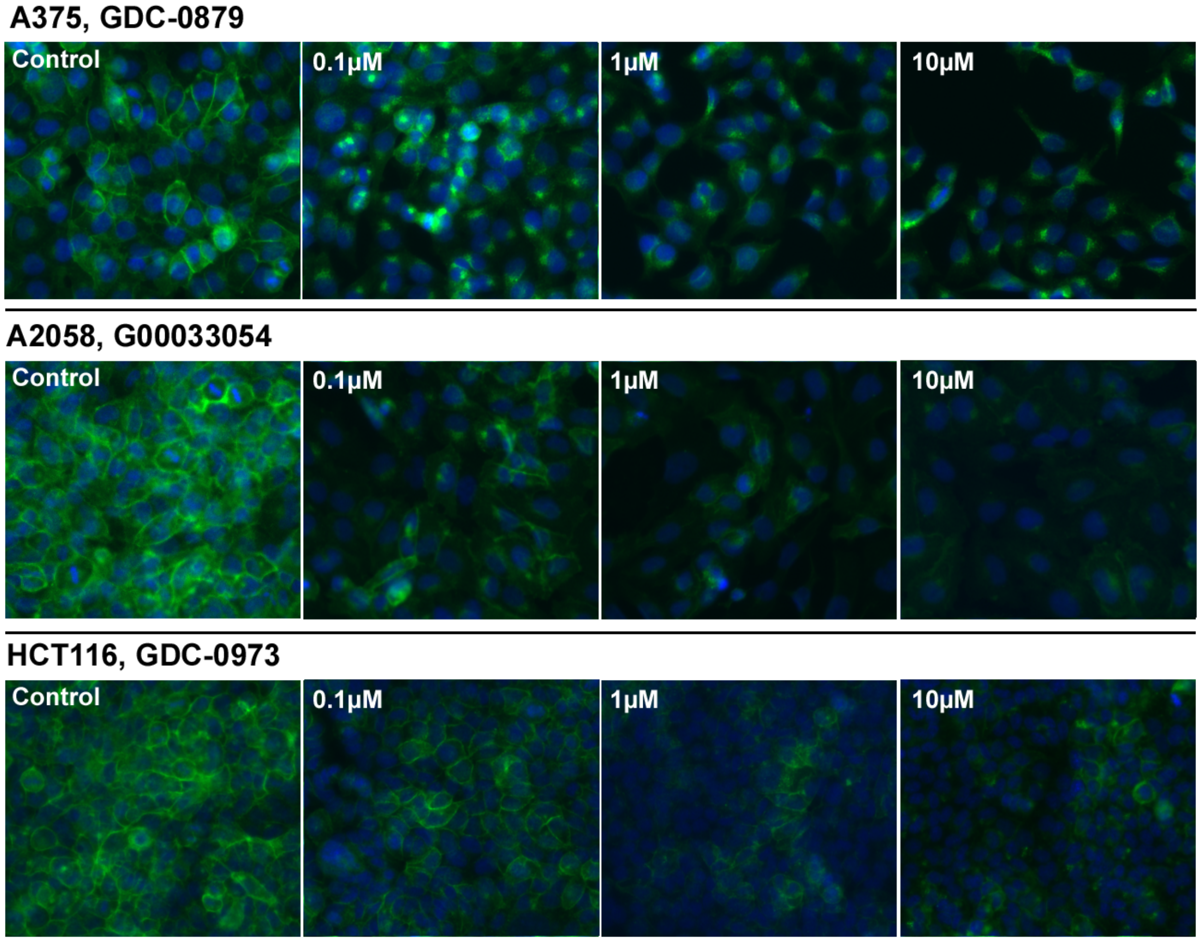

GLUT-1 immunoflurescence in vivo
Tumors treated for 7 days

METHODS

Glucose transporter immunofluoresence (In vitro): Cells were treated with drug, then were fixed in 4% paraformaldehyde, permeabilized with 0.2% Triton, blocked in 5% fish gelatin, incubated with Glucose transporter 1 antibody (Millipore Anti-Glut-1 07-1401) overnight, washed in PBS, incubated with secondary antibody Alexa 488 (Invitrogen #A11008) and Hoechst nuclear dye, washed in PBS, and then analyzed by fluorescent microscopy.

Glucose transporter immunofluoresence (xenograft sections): Xenograft tumors were excised and immediately frozen in isopentane cooled in liquid nitrogen, 7micron sections were cut, washed in PBS, permeabilized with 0.2% Triton, blocked in 5% fish gelatin, incubated with Glucose transporter 1 antibody (Millipore Anti-Glut-1 07-1401) overnight, washed in PBS, incubated with secondary antibody Alexa 488 (Invitrogen #A11008) and Hoechst nuclear dye, washed in PBS, and then analyzed by fluorescent microscopy.

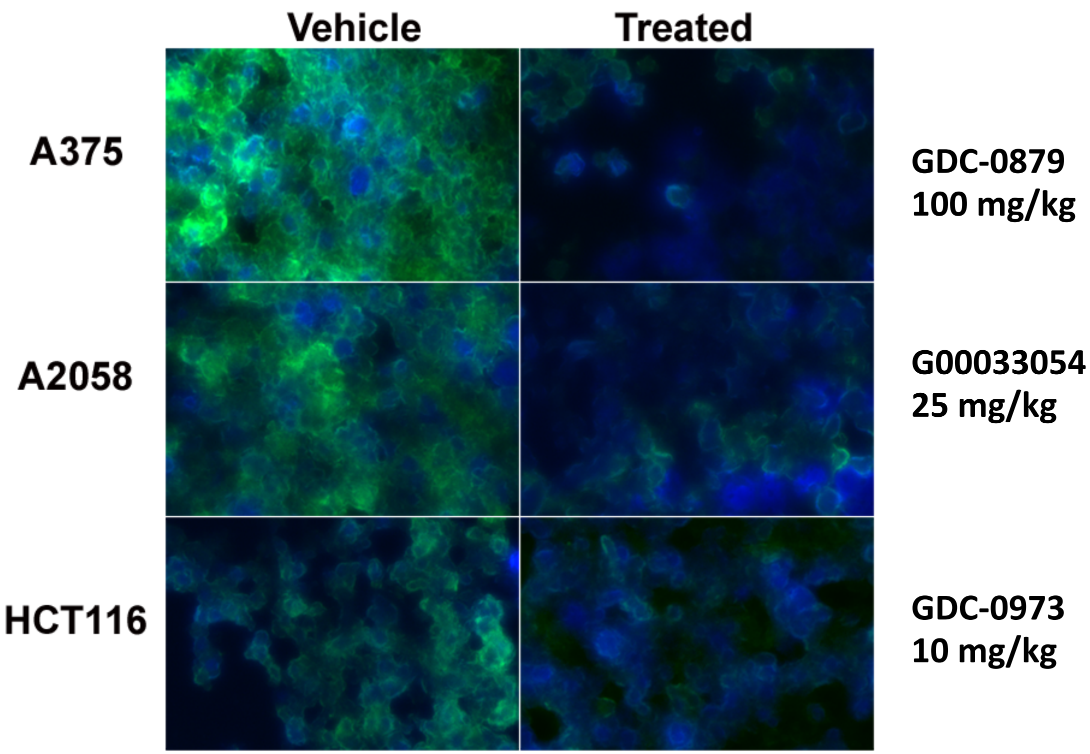

Supplement: Additional file 2 — Title: FDG in vitro and GLUT-1. Description: Evidence of drug action on cellular glucose uptake in vitro and in vivo for the same three models presented in Additional file 1. [file 2191-219X-2-35-S2.pdf]
