# Supplementary material for: The power of FDG-PET to detect treatment effects is increased by glucose correction using a Michaelis constant
Source: EJNMMI Res. 2012 Jun 27;2:35. doi: 10.1186/2191-219X-2-35 (PMC3472222; doi:10.1186/2191-219X-2-35)
Supplement: Additional file 3 — Title: Analytical derivations of the power functions. Description: Statistical power calculations: analytical derivation. [file 2191-219X-2-35-S3.pdf]

Additional File 3
Analytical derivations of the power functions

Power Calculations for K_i and $MR_{gluc}^{\max}$ based on the two-sample test of means.

Under the assumption of a Michaelis-Menten (MM) relationship between the FDG rate constant K_i and glucose $[glc]$ we derive the power functions of the two-sample z-tests based on K_i and $MR_{gluc}^{\max}$. We describe the conditions under which the power of the test based on $MR_{gluc}^{\max}$ dominates that of K_i, and show that the improvement occurs whenever the coefficient of variation (CV) in K_i is less than one. Additionally, we detail the unfavorable role of glucose variability on power. (In what follows, the superscripts c and t represent the control and treatment groups, respectively. Subject indices are omitted for notational ease.)

Let $\bar{K}_i^c, \bar{K}_i^t$ denote sample averages across n independent observations of K_i in the control and treatment groups, respectively. With a treatment effect of Δ, defined as the expected difference between observations in the control- and the treatment groups, for sufficiently large n, the test-statistic $Z_K = [(\bar{K}_i^c - \bar{K}_i^t) - \Delta]/\sqrt{(var(K_i^c) + var(K_i^t)/n}$ follows the standard normal distribution. As treatment effects are often expressed in relative terms, we write $\Delta = \delta K_i^c$ for some $\delta < 1$, where δ represents the proportion decrease in K_i due to treatment. For simplicity, we assume that the variances are the same in each group, *i.e.* $var(K_i^c) = var(K_i^t)$, but this requirement is not necessary. The power of a two sided test based on Z_k is then given by $P_k = \text{prob}(|Z_k| > z_{(1-\alpha/2)}|\Delta)$, where $z_{\alpha/2}$ is a standard normal quantile taken to ensure an α-level test. Thus, $\alpha = \text{prob}(|Z_k| > z_{(1-\alpha/2)}|\Delta = 0)$, the Type-I error rate.

Now, if $K_i > 0$ and $[glc] > 0$ are negatively correlated there exists constants $\gamma > 0$ and $\beta < 0$ such that the linear form $K_i \approx \gamma + \beta[glc] + \epsilon$ describes the correlation between K_i and $[glc]$. Here, ϵ is a zero-mean error process independent of $[glc]$ with variance σ_ϵ^2, and $[glc]$

is random with mean μ_g and variance σ_g^2. Thus, $var(K_i) = \beta^2\sigma_g^2 + \sigma_\epsilon^2$. In this case, with $\Phi(\cdot)$ the cumulative density function (CDF) of the standard normal distribution, the power of the z-test based on Z_K is

$$P_K = 1 - \Phi\left(z_{(1-\alpha/2)} - \frac{\delta K_i^c}{\sqrt{\frac{2\sigma_\epsilon^2}{n}(1+\beta^2\sigma_g^2/\sigma_\epsilon^2)}}\right) + \Phi\left(z_{\alpha/2} - \frac{\delta K_i^c}{\sqrt{\frac{2\sigma_\epsilon^2}{n}(1+\beta^2\sigma_g^2/\sigma_\epsilon^2)}}\right).$$

We stress that, for the above, the glucose process has the same mean (*i.e.* μ_g) in both the control and treatment groups. (The case when the treatment may alter μ_g is discussed in the main text.) We further note that the effect of glucose variability on P_K enters through the term $\beta^2\sigma_g^2/\sigma_\epsilon^2$. Thus, when $\beta^2\sigma_g^2/\sigma_\epsilon^2$ is non-negligible (relative to one), the term can be translated into an equivalent %-increase in sample size for equal power compared to the case when $\sigma_g^2 = 0$.

We arrive at essentially the same result if we assume that K_i follows a MM form corrupted by noise. That is, for some constants $MR_{gluc}^{\max} > 0$ and $Km > 0$, $K_i = MR_{gluc}^{\max}/(Km + [glc]) + \epsilon$, where ϵ and $[glc]$ are random processes as described above. Then, when the data is observed in a limited range around some glucose mid-point (say μ_g), $K_i \approx \gamma + \beta[glc] + \epsilon$, where $\beta = -MR_{gluc}^{\max}/(Km + \mu_g)^2$ (and some $\gamma > 0$). To illustrate the validity of the linear approximation in our setting, the left panel of Figure 1 shows 100 sample data points $\{[glc], K_i\}$ drawn from a MM model with parameters set near to the observed mean values across our studies (*i.e.*, $MR_{gluc}^{\max} = 45, Km = 130$, $[glc] \sim N(90, 25^2)$, and $\epsilon \sim N(0, .045^2)$; *c.f.* Results Section.) As seen in the left panel, the linear approximation closely follows the MM relationship in the data range, and the power curve for the MM model can be expected to closely follow P_K.

Under the assumption that the data is sampled from the MM model, the variance of $MR_{gluc}^{\max}$ equals $var(\epsilon(Km+[glc])) = \sigma_\epsilon^2((Km+\mu_g)^2+\sigma_g^2)$. Based on this, the power function P_M for testing equality of the maximal glucose uptake rate $MR_{gluc}^{\max}$ between the control and treatment groups based on sample means of $K_i(K_M + [glc])$ can be expressed as

$$P_M = 1-\Phi\left(z_{(1-\alpha/2)} - \frac{\delta K_i^c}{\sqrt{\frac{2\sigma_\epsilon^2}{n}(1+\sigma_g^2/(K_M+\mu_g)^2)}}\right)+\Phi\left(z_{\alpha/2} - \frac{\delta K_i^c}{\sqrt{\frac{2\sigma_\epsilon^2}{n}(1+\sigma_g^2/(K_M+\mu_g)^2)}}\right).$$

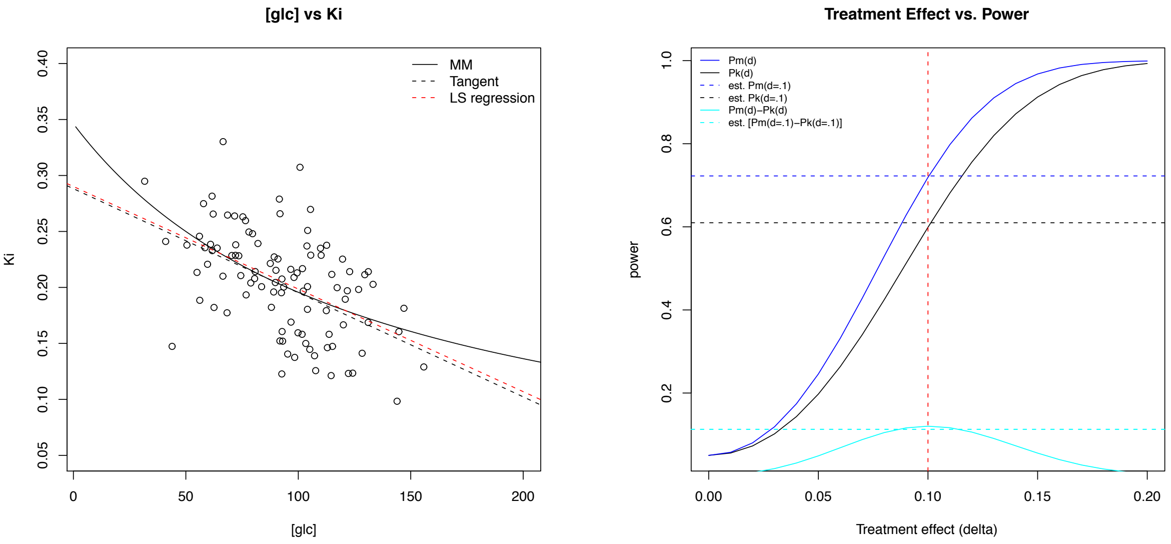

Figure 1: Left: Scatter plot of $[glc]$ versus K_i (see text for parameters.) The plot also shows the underlying MM process (solid black) along with tangent- (black dashed) and fitted (red dashed) LS-regression lines. Right: Power curves for $MR_{gluc}^{\max}$ (solid blue) and K_i (solid black) as a function of the treatment effect (δ). The dotted blue and black lines represent simulated values of P_M and P_K at $\delta = .1$. The theoretical difference in power (solid cyan) and the simulated difference at $\delta = .1$ (dotted cyan) are also shown.

To compare P_K and P_M we note that only the first CDF in the power functions will contribute to the power when $0 < \delta < 1$, *i.e.* when the treatment effect represents a $(100\times\delta)\%$ reduction in K_i. Therefore, P_K and P_M differ only through the terms $(1 + \beta^2\sigma_g^2/\sigma_\epsilon^2)$ and $(1+\sigma_g^2/(Km+\mu_g)^2)$ appearing in the denominators. We now substitute $\beta = -MR_{gluc}^{\max}/(Km+\mu_g)^2$ in the former, and obtain the following simple result: If $\sigma_\epsilon/K_i^c(\mu_g) < 1$, where $K_i^c(\mu_g)$ is the expected value of K_i^c evaluated at $[glc] = \mu_g$, then the power of the z-test based on $MR_{gluc}^{\max}$ is greater than that based on K_i. Thus, whenever the CV of K_i is less than one we get $P_M > P_K$. Now, in our retrospective analysis across the 66 studies, sample estimates of $\sigma_\epsilon/K_i^c(\mu_g)$ ranged from .1 to .5 (mean=.22), indicating the potential for improved power.

To illustrate the power result, the right hand side of Figure 1 shows P_K and P_M as a function of δ, where we have used the same set of parameters as those used to generate

the sample data (left) but with P_K and P_M evaluated for $n = 50$. With these parameter choices, CV = .22, and, as seen $P_M > P_K$. To test the accuracy of the depicted curves, we estimated the power at $\delta = .1$ using two-sided t-tests. Based on 4000 simulations, the estimated powers were $\hat{P}_M(.1) = 72.3\%$ and $\hat{P}_K(.1) = 61.0\%$, compared to the theoretical values of $P_M(.1) = 71.9\%$ and $P_K(.1) = 60.0\%$, respectively.
